# Nanocellulose hydrogels as bio-interface analogs for studying nanomaterial transport and accumulation

**DOI:** 10.64898/2026.02.02.703274

**Published:** 2026-02-04

**Authors:** Joshua Prince, Darryl Taylor, A-Andrew D. Jones

**Affiliations:** 1 Department of Civil & Environmental Engineering, Pratt School of Engineering, Duke University, Durham, NC 27708; 2 University Program in Materials Science & Engineering, Duke University, Durham, NC 27708,; 3 Thomas Lord Department of Mechanical Engineering & Materials Science, Pratt School of Engineering, Duke University, Durham, NC 27708

**Keywords:** Biointerface materials, nanoparticles, antibiotic recalcitrance, transport phenomena

## Abstract

Nanomaterials have been proposed as drug delivery vehicles to enhance targeting and efficiency of traditional and novel therapeutics and have subsequently been studied for potential ecotoxicity. Previous studies have identified size, surface charge, and volume exclusion as factors that influence nanomaterial diffusion and retention. However, there is little accepted or successful quantification of how these parameters influence nanomaterial penetration relative to biological adaptation and biological response. Part of the challenge is the response of living biological interfaces to many of these nanomaterial delivery vehicles and nanosized drugs. This study aimed to emulate key physicochemical barriers to diffusion found in living biomaterials by developing a tunable, synthetic hydrogel. Through the controlled exposure of 150 kDa and 2 MDa nanodextrans with neutral and negative surface charge, we evaluated the system’s ability to emulate three core physicochemical features often implicated in biofilm-associated transport resistance: size exclusion, charge interactions, and volume exclusion. We demonstrated a 30% statistically significant decrease in partition coefficients for 2 MDa nanodextran from 150 kDa nanodextran, confirming the ability of the nanocellulose-based microcaps to mimic the permeability of hydrated biomaterial matrices. These findings reflect patterns observed in, for example, living biofilm studies, where size-based diffusion hinderance is commonly reported, but charge-based interaction and volume exclusion are more context-dependent. This controllable system can be coupled with *in silico* modeling to understand interfacial transport phenomena for nanomaterial-biomaterial interactions.

## Graphical Abstract

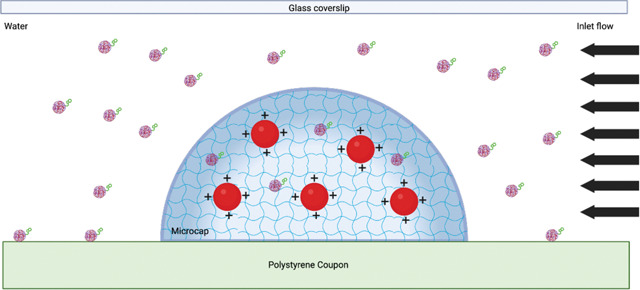

## 1. Introduction

Biological interfaces, like microbial cells and biofilms, are more complex than engineered porous membranes[1] and subsequently the transport relationships are more complex. For example, the liquid-surface biointerface typically contains soft-layers comprised of fats, sugars, and proteins of various charges [2]. The interior of the biointerface exhibits heterogenous porosity and tortuosity [3], heterogeneous mechanical compliance, heterogenous hydrophilicity, and heterogeneous charge distribution due to the presence of cells and extracellular components [4]. These complex structures increase the difficulty of decoupling the impacts of charge, size, geometry, and chemistry on the transport of novel drug and drug delivery vehicles through these biological interfaces. In microbial biointerfaces, like bacterial biofilms [5–8], this complexity is increased by the dynamic response that can occur at similar time scales to the time for total penetration. The dynamic response from bacteria can include extracellular matrix production of polymers like alginate, nanocellulose, proteins like flagellin [9, 10], and secretion of extracellular DNA. These properties contribute to enhanced virulence [11] and treatment failure [12] in diseases such as cystic fibrosis. Consequently, new therapeutic strategies, including the use of nanomaterials as drug delivery vehicles, have been proposed to enhance penetration and efficacy within biofilms[13–15].

These new therapeutic strategies will come with their eventual environmental dissemination. While engineered nanomaterials cannot be studied as a uniform class[16], experimental and computational platforms that account for their fate and transport must be created [17–21]. Periphyton biofilms are the primary sink for nanomaterials in estuary environments [22]. The primary factors which effect ENM ecotoxicological parameters such as toxicity and bioaccumulation for a given ENM-biofilm-environment system can be evaluated. What emerges is a set of four pairwise factors of the biofilm-ENM system of interest which shapes bioaccumulation: size (particle/pore) [23–29], charge (surface/ECM) [24, 25, 27, 30–32], chemistry (surface/ECM) [25, 31, 33], and hydropathy (surface/ECM) [27, 30, 34].

Previous studies on the impact of mechanical stress on biointerfaces have used alginate since it is an essential component of some biofilms and has facile preparation [35–38]. However, alginate has 1 −2 orders of magnitude higher storage and loss moduli than natural biofilms [37]. Additionally, alginate has different chemical absorption properties, specifically it is less able to absorb divalent metallic cations than natural biofilms [37]. We have previously shown the nanocellulose preparation used here has mechanical and certain chemical properties in range with natural biofilms [39]. Nanomaterial exclusion from biofilms has been proposed to be a function of the various heterogeneous matrix components alginate, nanocellulose, proteins; the matrix size; commensal phage; bacterial cells, particularly their charge, and the channels in the biofilm [4, 30, 40]. As heterogeneity is difficult to achieve synthetically, this may require systematic study. For example, the importance of alginate on antibody binding in *P. aeruginosa* biofilms was discovered through adding alginate back alginate deficient mutants. Recent work on diffusion in alginate showed the impact of matrix cross-linking on size-exclusion [41].

We propose the biofilm matrix and cells with their respective charges can be studied with synthetic systems. We synthesized microcaps from nanocellulose that are modified with divalent calcium ions as the biofilm matrix. We added neutral and charged microspheres to represent cells and their respective binding affinities. We tested the ability of charged and neutral nanodextran of different molecular weights to accumulate into the defined nanocellulose matrix.

This study tested the ability of a microcap synthesis method to replicate the important effects of a biofilm on species diffusion: (a) the size-effect, (b) the volume-exclusion effect, and (c) the attachment effect. These important biofilm effects were mapped on to three hypotheses. We hypothesize that if the size-effect is replicated that larger diffusing species would accumulate at lower concentrations in the matrix. We hypothesize that if the volume-exclusion effect is replicated, that diffusing species would accumulate at a lower concentration in a microcap with impermeable particles compared to one without. We hypothesize that if the attachment effect is replicated that diffusing species would accumulate at a higher concentration in the microcap when attachment sites are present. Biofilms have distinct porosity profiles based on environmental conditions [42, 43], distinct matrix components based on nutrient conditions and external threat [36, 43], and distinct cell populations based on age and nutrient conditions. This study and platform may aid in understanding how each component contributes to overall accumulation.

## 2. Methods

**Figure 1:**
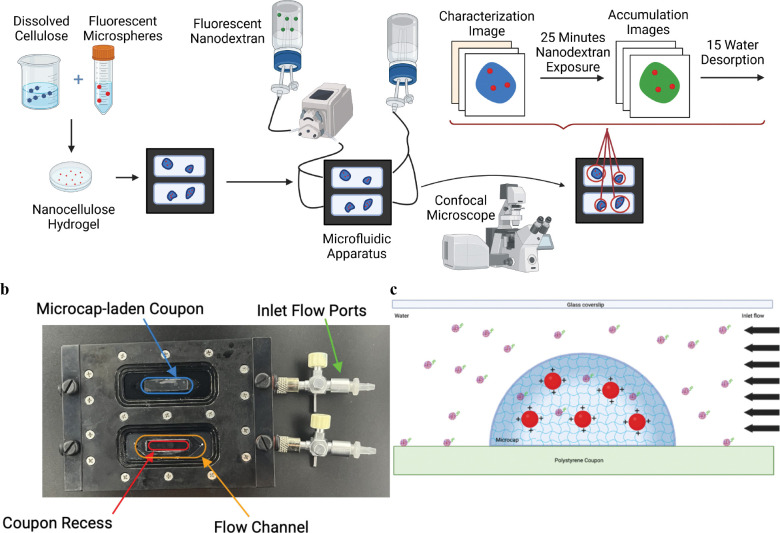
A. Experimental protocol for testing diffusion into hydrogel. B. Experimental Apparatus Diagram C. Artistic representation of microcap. Hydrogels were made as described in [39]. After centrifugation, microspheres were added. Resultant, A flow cell (b) from Biosurface Technology was seeded with hydrogel inserts along the coupon recess/imaging window. Red circles represent microspheres. Microspheres with amine-coating represented. Nanodextran with no modification shown.

The goal of this study is to test the ability of a synthetic biomaterial to replicate the important effects of a biofilm on species diffusion. We leveraged our recently developed nanocellulose hydrogel that has closer mechanical stiffness to natural biofilms. We embedded the biofilm with charged and non-charged microspheres to test volume-exclusion and attachment and charge based exclusion. We used two sizes of nanodextrans to test size exclusion. We used standard optical tools for examining biofilms to measure the effects of the tested parameters, Figure 1.

### 2.1 Microcap Design and Nanodextran Information

To generate the microcaps, hydrogels were used as the hydrated mesh domain to achieve a size-effect. Hydrogels have been used in prior studies to simulate the extracellular matrix of the biofilm [41]. The hydrogel platform chosen for our system was nanocellulose hydrogels. We recently developed a nanocellulose that better matched the mechanical storage and loss moduli [39], that is also easily chemically modifiable for which future studies could choose to utilize for a method of generating attachment sites independent of microspheres.

Fluorescent polystyrene microspheres (Spherotech | Lake Forest, IL) ranging from 0.7–0.9 microns in diameter were used to simulate bacteria and achieve a volume-exclusion effect. Three distinct microspheres, each with distinct microsphere surface chemistry, were used: no surface modifications (Plain) (Prod# FP-0862–2), carboxylate-modified (Carboxyl) (Prod# FP-0862–2), and amine-modified (Amino) (Prod# FP-0862–2). The carboxylate and amine-modified microspheres were coated with these functional groups, which are either negatively or positively charged at neutral pH. These charged microsphere surfaces were designed to only act as attachment sites for the diffusing species if the diffusing species was oppositely charged. The microspheres were fluorescently labelled with a vendor proprietary fluorescent tag.

Nanodextrans conjugated to FITC (fluorescein isothiocyanate) were used as the diffusing species for the system (TdB Labs, Uppsala, Sweden). Two nanodextran molecular weights used were 150 kDa and 2,000 kDa, on the premise these molecules would have different sizes, to test the size-exclusion effect. The fluorescently labelled nanodextrans were chemically modified to have either carboxymethyl (CM) groups with negative charge (Prod# FITC-CM-dextran-150), diethylaminoethyl (DEAE) groups with positive charge (Product # FITC-DEAE-dextran-150), or no modification at all (Dx) (Prod# FITC-Dx-150 and Prod# FITC-Dx-2000). The use of charged nanodextrans and charged microsphere surfaces was designed to produce the attachment effect.

### 2.2 Nanocellulose Solution Synthesis

A nanocellulose synthetic biofilm was generated as described in [39]. Briefly, 4.00 ± 0.05 g of dry cellulose powder (CAS#9004–34-6 | Sigma-Aldrich | Burlington, MA) and slowly mixing it with an ionic liquid solution composed of 7.00 ± 0.05 g sodium hydroxide (CAS#1310–73-2 | Sigma-Aldrich | Burlington, MA), 12.00 ± 0.05 g urea (CAS#57–13-6 | Sigma-Aldrich | Burlington, MA), and 81.00 ± 0.05 g of reverse-osmosis filtered (RO) water and adding it to a 125 mL Erlenmeyer flask. The resulting suspension was mixed using an inert stir bar at 500 rpm until it was homogenous and consistently cloudy. The suspension was then submerged in an ice-isopropyl alcohol bath with a temperature ranging between −10 and 0 °C while still being stirred. After more than 1 hour of stirring and the dissolution of the cellulose, the liquid was separated into two 50 mL centrifuge tubes. The tubes were centrifuged at 10,000 RPM (rcf=13,751) for 10 minutes while maintaining a temperature of 22 °C. After centrifugation, the supernatant was decanted and saved as nanocellulose solution, with any settled solids discarded.

### 2.3 Nanocellulose-Microsphere Solutions

For each batch of nanocellulose solution made, four different nanocellulose-microsphere solutions were made: one with Plain microspheres, one with Carboxyl microspheres, one with Amino-microspheres, and one with no microspheres. These nanocellulose-microsphere solutions were prepared by mixing 5.0±0.1 mL of nanocellulose solution with 50±0.1 μL of 1wt% microsphere solution in the well of a 6-well culture plate. Once all four nanocellulose-microsphere solutions were prepared for each nanocellulose batch on the same plate, the plate was covered, wrapped in aluminum foil, and mixed for 24 hours on a shaking plate at 250 RPM at room temperature ranging from 22–26 °C. After 24 hours of mixing, the resulting nanocellulose-microsphere solutions were stored at 4 °C.

### 2.4 Microcap and Flow Cell Preparation

With the nanocellulose-microsphere solutions prepared, a 5 mL syringe affixed with a 27G nozzle was used to fill the nozzle with nanocellulose-microsphere solutions. The nanocellulose solution was then extruded through the nozzle using the syringe. Once a small amount of nanocellulose solution was extruded, priming the nozzle, the nozzle would then “leak” the solution semi continuously. The nozzle tip was then repeatedly (between 10–30 times) pressed onto the surface of a polycarbonate coupon, with a microscopic amount of hydrogel being left behind on the coupon as residue with each press. The coupon then sat at room temperature in a plastic petri dish for greater than 48 hours to dry with no plastic cover but aluminum foil cover, which prevented contamination and fluorescent overexposure, but allowed for air flow. This let the hydrogel residues to dry onto the coupon and form microcaps. Before an experiment, the microcap covered coupon was loaded into a flow cell (Prod# FC 275-AL | BioSurface Technologies | Bozeman, MT, USA), where the flow cell chamber containing the coupon was filled with reverse-osmosis water first to rehydrate the microcap. Almost immediately after, the chamber was then filled with a 5 μM solution of Calcofluor White (CW) stain dissolved in water (Cat# 29067 | Biotium | Fremont, CA, USA). The fluorophore would then slowly diffuse into the microcaps, allowing for fluorescent imaging of the hydrogel domain of the microcap.

### 2.5 Nanodextran Exposure and Imaging Procedure

Once the flow cell was loaded with coupons covered with CW-stained microcaps, the flow cell was then connected to a microfluidic apparatus with two inlets: reverse-osmosis filtered (RO) water and 30 mg/L fluorescently labelled-nanodextran dissolved in Dulbecco’s phosphate buffer solution (PBS) (Figure 1). The flow cell was placed on a confocal laser scanning microscope, which allowed for real-time 3-D fluorescent imaging of the microcaps.

Each experiment involved a two-phase procedure: characterization and accumulation. To perform an experiment, the flow chamber was flushed with 1 mL/min RO water during the characterization phase. While the flow chamber containing the microcap-covered coupon was flushed with 1 mL/min RO water, the microcaps on the coupon were checked to determine if there were three microcaps less than 250 microns in diameter (all tests had at least three). A z-stack, or a series of microscope images taken at close, regularly spaced focal lengths to reconstruct a 3-D image of a sample, was taken of three microcaps on each coupon (with the three smallest microcaps usually chosen). Field of view size was adjusted for each microcap to take up the entire image. Nyquist sampling was used to select z-section thicknesses and image resolution. These z-stacks were set up to image three features in each microcap: the hydrogel mesh by imaging the CW stain (Laser: 405 nm/Detector Range: 410–546 nm), the impermeable microspheres (Laser: 561 nm/Detector Range: 585–700 nm), and the presence of FITC (Laser: 488 nm/Detector Range: 410–546 nm). In addition to measuring the presence of FITC, this same signal was also used to determine the location of the coupon within the image, as the coupon reflected green light at its surface. This first image before any FITC was added to the system will be referred to as the characterization image.

After the characterization image of each of the microcap of interest, the microcaps were exposed to a continuous stream of nanodextran during the accumulation phase of the experiment. This was done by flowing 1 mL/min of the 30 mg/L nanodextran dissolved in PBS into the flow chamber continuously for 24 minutes. After this continuous exposure to a constant concentration of nanodextran, the flow in the chamber was stopped, and the microcaps re-imaged using the same parameters as those for the characterization image, producing an accumulation image for each microcap. This was done to quantify two things: the concentration of nanodextran (via the proxy measurement of FITC-signal) in the water immediately surrounding each microcap, and the concentration of nanodextran within each microcap.

During accumulation phase of each experiment, time-lapse imaging of a single-z-plane within one of the imaged microcaps was taken to monitor if the system reached equilibrium during each phase (which all were confirmed to reach).

### 2.6 Experimental Design

**Figure 2:**
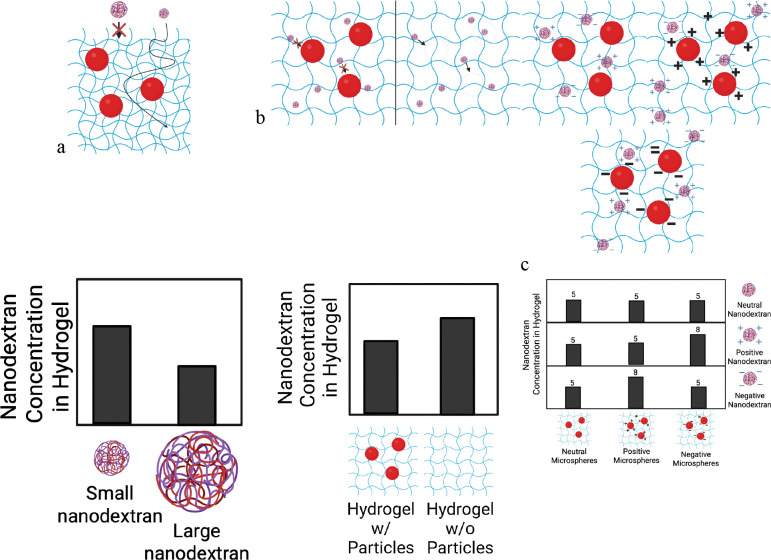
**(a-c)**Diagrams of hypothesized size-exclusion effect, charge based attachment effects, and volume exclusion effect. **d-e** Hypothesized outcomes of nanodextran concentration in hydrogels based on the given effect.

To test the functional properties of these microcaps, three predicted experimental effects were tested to determine the capacity of these microcaps to replicate the biofilm features of interest, Figure 2. The size-exclusion effect was tested to determine if a hydrated mesh was formed. The volume-exclusion effect was tested to determine if the embedded microspheres were diffusing species impermeable. The attachment effect was tested to determine if the diffusing species were immobilized via opposite charge interactions with the surface of the microspheres, indicating diffusing species attachment sites.

To test for the size-exclusion effect, the two different sized nanodextrans were used: 150 kDa and 2,000 kDa. The 2,000 kDa nanodextran was expected to have a larger particle size than the 150 kDa case. This was tested and confirmed using Nanoparticle Tracking Analysis and Dynamic Light-Scattering measurements, shown in Table 1. While the measured sizes were similar sizes via NTA and DLS, we used them as it well known that while designed for specific sizes, environmental nanomaterials are never pristine. These nanodextan molecular weights were chosen because they have been used in biofilm penetration studies as both small and large sized nanomaterials [23, 25, 44, 45]. Additional details on nanoparticle characterization are provided in the Supplementary Information.

Since hydrated meshes such as a biofilm extracellular matrix and a hydrogel are comprised of disorganized, overlapping biopolymer chains, they can allow particles to diffuse through them, but only up to a certain size. As the particle reaches a characteristic “mesh size”, less of the volume within the hydrated mesh network is available for it to occupy. Once the particle reaches a critical size, no amount of particle can accumulate within the matrix. Thus, as particle size increases, the equilibrium concentration it reaches within a hydrated matrix decrease, as less volume is available for the particle to permeate into. Hence, a decrease in diffusing species concentration with particle size within the matrix at equilibrium shows the presence of a size-exclusion effect in the microcap, which in our system is likely indicative of the formation of a hydrogel. Microcaps prepared using identical conditions were used in size-exclusion experiments.

To test for the volume-exclusion effect, hydrogels were prepared either with microspheres within them or without microspheres. The microcaps without microspheres within them would be expected to reach a higher concentration of nanodextran if the volume-exclusion effect was observed. The reasoning for this is similar to the reasoning for the size-exclusion effect: certain portions of the microcap volume, specifically the volume occupied by the microspheres, would be unavailable for the nanodextran to occupy, leading to a lower concentration overall in the microcap. Identical nanodextrans were used in volume-exclusion experiments.

To test for the attachment effect, hydrogels embedded with three different microspheres were prepared and tested against three different nanodextrans. The different microspheres tested were no surface functionalization (Plain, neutrally charged surface), amine-functionalization (Amino, positively charged surface), and carboxyl-functionalized (Carboxyl, negatively charged surface). The different nanodextrans tested were no chemical modification (None, neutrally charged), carboxymethyl-modified (CM, negatively charged) and diethylaminoethyl-modified (DEAE, positively charged). Since we only expect oppositely charged combinations to lead to attachment between microsphere surface and nanodextran, we expect to see significant increases in nanodextran concentration within the microsphere only for the combination of CM-modified nanodextran accumulating in microcaps with amine-modified microspheres, and DEAE-modified nanodextran accumulating in microcaps with carboxyl-modified microspheres. Two different control cases are considered for each of these combinations: (a) charged nanodextran and uncharged microspheres, and b) uncharged nanodextran and charged microspheres. Decreases in nanodextran concentrations within the microcap would be expected in both cases compared to the oppositely charged nanodextran/microspheres case.

### 2.7 Image Analysis

To determine nanodextran concentration for each z-stack image taken, each pixel was assigned to be in one of four spatial domains in the image: (1) the water domain (liquid domain $\Omega_{L}$), (2) the hydrogel domain (interstitial domain $\Omega_{I}$) (3) the microsphere domain ($\Omega_{O}$), and (4) the coupon domain (solid domain $\Omega_{S}$).

This was done by segmenting the hydrogel using the CW signal, the microspheres using the AF594 signal, and the coupon using the FITC-signal in each image, and assuming all remaining pixels were within the water domain. The total microcap domain was the union of the microcap and microsphere domains.

The microspheres were segmented using an Otsu threshold on the AF594 signal in MATLAB. The hydrogel domain was segmented in ImageJ using a Otsu threshold, followed by a region-filling algorithm (CW-stain only penetrated ~10 microns into hydrogel), followed by an algorithm for discarding very small filled regions. The coupon was segmented using an edge-detection algorithm along the z-direction. An important note on this process was that coupon segmentation was not perfect. The coupon segmentation algorithm was designed to favor a pixel as coupon as opposed to water or hydrogel on purpose, since the accuracy of the water and hydrogel signal was more important than the coupon signal.

With each pixel assigned to a domain, the average value of the nanodextran/FITC signal in each domain in the microcap was quantified. Rather than use average FITC-signal in the microcap, $[FITC{]}_{\mathrm{microcap}}$, directly, the ratio of the FITC-signal in the microcap to the FITC-signal in the water, $[FITC{]}_{\mathrm{water}}$, or the nanodextran-microcap partition coefficient, $K_{P}$, was calculated for each microcap’s accumulation image, Equation 1. This ensured any variation in FITC concentration and signal within the flow cell was normalized between microcaps.

Equation 1: Microcap Partition Coefficient

$$K_{P}=\frac{[\mathrm{FITC}{]}_{\mathrm{microcap}}}{[\mathrm{FITC}{]}_{\mathrm{water}}}$$

The MATLB code for image analysis is included as Supplementary Information and is available via Github.

### 2.8 Statistical Analysis

For each nanodextran-hydrogel combination tested, two types of replicates were used, all on the hydrogel side: batch replicates and microcap replicates. Three batches of each hydrogel tested (no microspheres, plain microspheres, carboxyl-microspheres and amine-microspheres) were generated, leading to 12 different hydrogels with three batch replicates each. For each batch, one coupon covered with microcaps was generated for each nanodextran tested, leading to 33 different coupons. On each coupon, three microcap replicates were tested on each. This led to a total of 99 microcaps tested.

To compile the data for each experimental condition (nanodextran-microcap combination), the nanodextran-microcap partition coefficient for each microcap tested was first found (see Image Analysis for how this was calculated). This value was then averaged for the three microcaps on each coupon tested, as these replicates were determined to be technical replicates, which warrant averaging together for the purposes of statistical analysis. This left three nanodextran-microcap partition coefficients for each experimental condition, each one representing a different hydrogel batch. These three nanodextran-microcap partition coefficients comprised the sample for each experimental condition.

For each set of experimental conditions compared for each hypothesis, the three nanodextran-microcap partition coefficients for each batch were compared to each other using Welch’s one-way t-tests using an alpha of 0.05. Welch’s test was used since variances were not assumed to be equal. One-way tests were used since only an effect in one direction would constitute evidence for each hypothesis. All samples passed the Shapiro-Wilks test for normality.

## 3. Results

The microcaps generated using this synthesis procedure were tested for their ability to replicate the core features of a synthetic biofilm. These included size-exclusion effects, volume exclusion effects and attachment effects. Different combinations of microcaps and diffusing nanodextran were used to test for these effects through equilibrium nanodextran partition coefficients in the microcaps.

### Testing for Hydrated Mesh Property via the Size Effect

**Figure 5:**
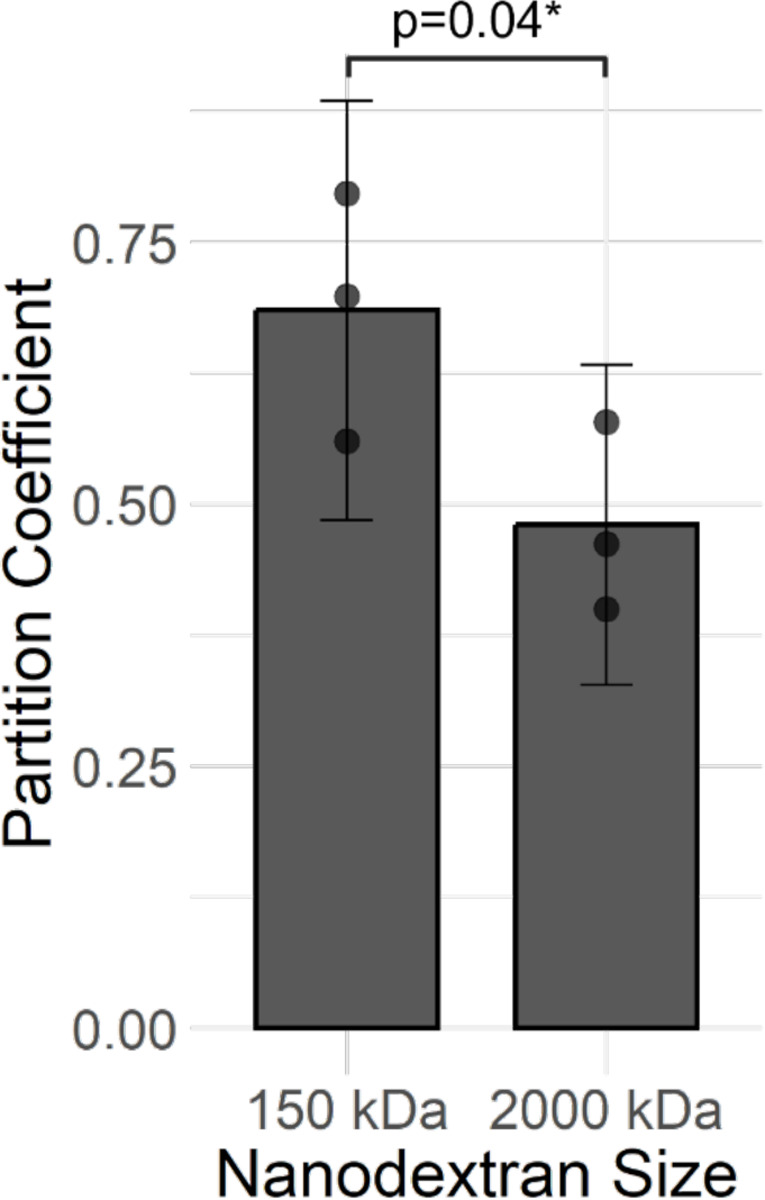
Size-Exclusion Effect Test for Verifying the Hydrogel Mesh Property of the Synthetic Biofilm System. Error bars represent 95% confidence intervals on the sample. P-value represents results from one-tailed Welch’s t-test. Experiments used 4 wt% hydrogels loaded with 0.01 wt% plain polystyrene microspheres with neutrally charged nanodextrans. Partition coefficient represents nanodextran-microcap partition coefficient.

To test for the size-exclusion effect, nanodextran-microcap partition coefficients for microcaps with plain microspheres were compared between a 150 kDa nanodextran and a 2,000 kDa nanodextran. The results, as shown in Figure 5, show a statistically significant size-exclusion effect in the hydrogel mesh was observed.

### Testing for Impermeable Sub-volume via the Volume-Exclusion Effect

**Figure 6:**
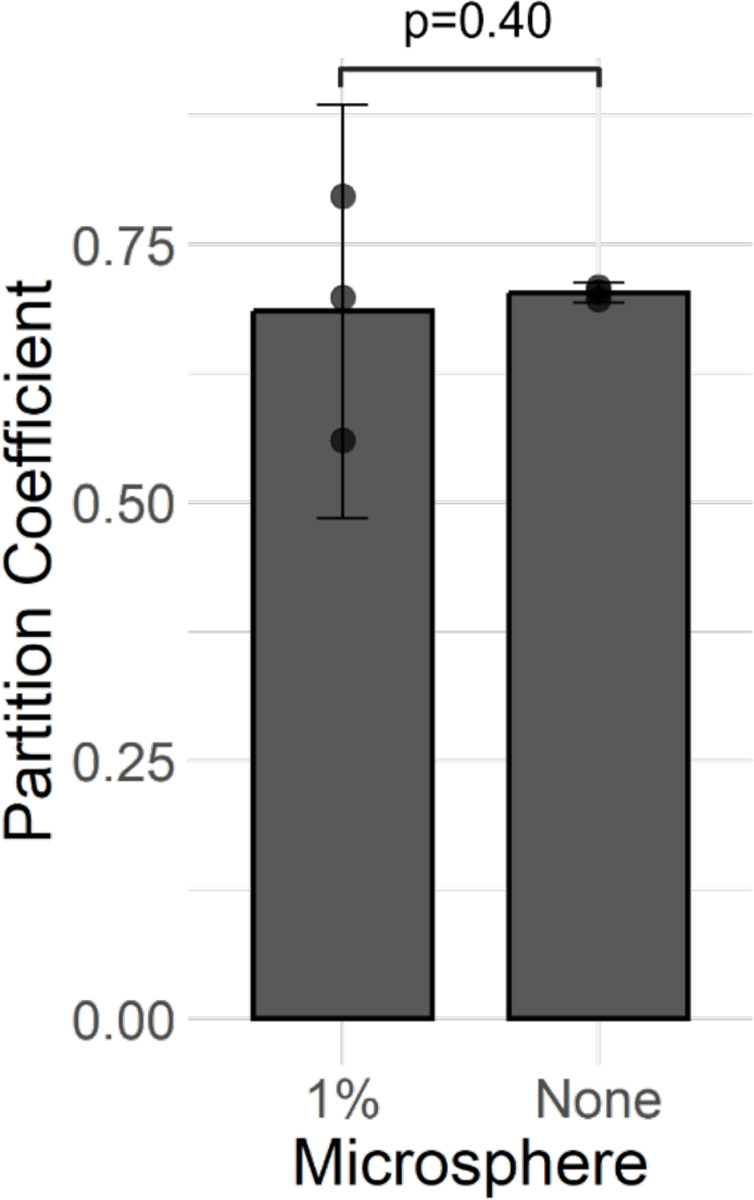
Volume-Exclusion Effect Test for Verifying the Impermeable Subdomain Property of the Synthetic Biofilm System. Error bars represent 95% confidence intervals on the sample. P-value represents results from one-tailed Welch’s t-test. Experiments used 4 wt% hydrogels loaded with variable wt% plain polystyrene microspheres and neutrally charged, 150 kDa nanodextran. Partition coefficient represents nanodextran-microcap partition coefficient.

To test for the volume-exclusion effect, nanodextran-microcap partition coefficient for 4 wt% hydrogel microcaps embedded with either no microspheres or 0.01 wt% plain microspheres were compared using a neutrally charged, 150 kDa nanodextran. The results, as shown in Figure 6, show no volume-exclusion effect due to the presence of the microspheres was observed.

### Testing for Attachment Sites via the Attachment Effect

**Figure 7:**
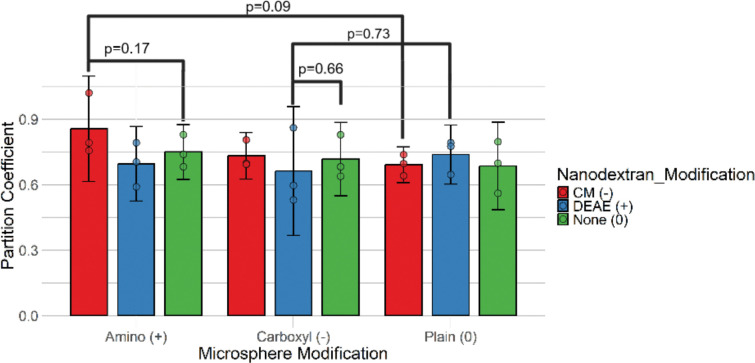
Attachment Effect Test for Verifying Presence of Attachment Sites in the Synthetic Biofilm System. Error bars represent 95% confidence intervals on the sample. P-values represent results from one-tailed Welch’s t-test. Experiments used 4 wt% hydrogels loaded with variable 0.01 wt% plain-, carboxyl- and amine-modified polystyrene microspheres and neutrally, positively or negatively charged, 150 kDa nanodextrans. Partition coefficient represents nanodextran-microcap partition coefficient.

To test for the attachment effect, 4 wt% hydrogels embedded with 0.01 wt% neutrally, negatively, and positively charged microspheres were tested against neutrally, negatively, and positively charged 150 kDa nanodextrans, shown in Figure 7. Opposite charge combinations were tested for increases in nanodextran-microcap partition coefficients compared to both the same charged nanodextran and uncharged microspheres and the uncharged nanodextran and same charged microspheres. No statistically significant attachment effect was observed across all comparisons. A non-statistically significant attachment effect was observed for the microcaps embedded with amine-modified microspheres with a carboxymethyl-modified nanodextran for comparisons to both control cases.

## 4. Discussion

This research sought to create a synthetic biofilm system for use studying species transport in biofilms. To accomplish this, nanocellulose hydrogel microcaps loaded with polystyrene microspheres were developed to proxy bacterial biofilms in the features of a hydrated mesh, an impermeable subdomain, and presence of attachment sites. The replication of these features in the generated microcaps were tested for by quantifying an important experimental effect for each feature: the size-exclusion effect for verifying the hydrogel mesh, the volume-exclusion effect for verifying the impermeable subdomain, the attachment effect for verifying presence of attachment sites.

Experimental results indicated that the size-exclusion effect was verified, and therefore the hydrogel mesh feature of the microcaps. However, the attachment effect and the volume exclusion effect were not observed. This indicates that the microcaps as currently designed are unable to replicate the key biofilm properties needed to perform transport studies of an impermeable subdomain and presence of attachment sites.

Size-effect agrees with prior literature on chemical species diffusion in single bacterial species biofilms [23, 46]. However, evidence of this effect is limited in multispecies biofilm literature on nanoparticles [47]. This is most likely due to effects of agglomeration, dissolution and changes to the nanoparticle biopolymer corona in multispecies biofilms studies having strong effects on effective nanoparticle size during diffusion [48, 49]. This shows the need for studies with more controls over nanoparticle stability in multispecies biofilm transport studies.

A few distinct hypotheses could explain why the presence of microspheres and the use of chemically modified microsphere surfaces and nanodextrans did not elicit the effects of volume-exclusion and attachment. One is the use of a 0.01 wt% microsphere percentage within the hydrogels was too low to observe the anticipated effects. If little volume is made impermeable due to low concentration of microspheres, then the volume-exclusion effect would be anticipated to be small and below any statistically significant threshold. In addition, too low of a concentration of microspheres would mean there are very few sites for nanodextran attachment, which would also predict a small attachment effect again below any statistically significant threshold. Another possibility is that nanodextran can penetrate and diffuse into the polystyrene microspheres. While there is evidence for adsorption onto the surface of polystyrene [50], there is less evidence for penetration and diffusion into polystyrene microsphere. However, no experimental evidence is offered in this research proving or disproving this hypothesis.

For the attachment effect, another possibility is that the charged microsphere surfaces do not act as attachment and immobilization sites for its oppositely charged nanodextran. This could be due to a variety of reasons including the charge-charge interactions not being strong enough to immobilize the nanodextran, the microsphere or the nanodextran not being charged species as anticipated, or even that the immobilization of the nanodextran does not lead to higher levels of nanodextran to accumulate into the hydrogel to balance the chemical potential gradient. While it is possible attachment does occur in these systems but too slowly to observe in this experiment, direct charge-charge interactions are the strongest intermolecular forces in chemistry, which would make for quick attachment, making this possibility seem unlikely. This lack of a clear effect of charge is common in the scientific literature on diffusion of chemical species in hydrogel matrices such as biofilms [41, 44, 45, 51, 52]. Future experiments on this system could include testing the accumulation and desorption of ions instead of nanomaterials as these have been shown to matter in the biofilm matrix [53].

Assuming the hypothesis that the non-observed effects were primarily due to a low concentration of microspheres within the microcaps, further experimental work could replicate these experiments with higher concentrations of microspheres and look for the same effects on attachment and volume-exclusion. Further evidence for the size-exclusion effect could be found by comparing current results to higher weight percentage nanocellulose hydrogels, which theoretically lead to smaller critical mesh size, which would be anticipated to cause lower nanodextran concentrations in these higher weight percentage microcaps [23, 54].

The data presented here does not lend itself to diffusivity calculations common in the literature. However, Bryers and Frummond showed lumped parameter diffusivity calculations are inaccurate for describing transport in biofilm [55]. The data can be compared to bioconcentration factors (BCFs) which have been widely reported in ecotoxicological studies of engineered nanoparticles effects on environmental biofilms. Since estimates for BCFs in ecotoxicological studies are usually only order of magnitude estimates, logarithmic BCF values (pBCF) will be used here to compare BCFs between different experiments [56–58]. pBCFs will be calculated from dimensionless BCFs on a mass-by-mass basis (μg ENM/g biofilm divided by μg ENM/g water) using the wet weight of the periphytic biofilms when possible. Since periphytic biofilms are hydrated in the environment, wet weights are used to give a more intuitive sense of the extent of the increase in concentration of ENM that would be seen in an environmental system. If only dry-weight/AFDM BCFs are reported, wet-weight BCFs will be estimated by assuming a 100:1 ratio of biofilm wet mass to biofilm dry mass. Finally, many studies for the bioaccumulation of ENM quantify total metal uptake, not ENM specific uptake. Unless controls for dissolution or specific quantification of ENM is stated, all BCFs reported here will reflect this, limiting their representation of “true” ENM BCFs. Since most studies do not report detailed kinetic analysis of ENM bioaccumulation, characteristic times, $t_{C}$, of bioaccumulation will be estimated when possible. Estimations of these are mostly qualitative, representing approximately the time necessary to reach concentrations 50% of equilibrium concentrations.

The data in this study are 2 – 10 times less than the pBCF reported in literature, Table 2. While this hydrogel matches mechanical properties better than alginate, many biofilms contain both alginate and nanocellulose polymers. Lastly, biofilms are much more than cells or beads distributed in a hydrogel mesh, they also contain channels filled with pore water [55]. Equation 2 shows that if the amount of nanoparticles in the pore water is in equilibrium with the surrounding water, the BCF in a porous biofilm will be less than an equivalent amount of nanomaterials being added to a fixed amount of biofilm mass without. Equivalently, the pBCF of a biofilm with pores will be greater than one without for the same amount of substance added to the biofilm, by a factor of log2 for biofilm of equal weight to water.


Equation 2 $$\frac{\frac{m_{nDx,b}\;+\;m_{nDx}}{m_{b}\;+\;m_{w}}}{m_{nDx}/m_{w}}=\frac{\frac{m_{nDx,b}\;+\;m_{nDx}}{m_{b}\;+\;m_{w}}}{m_{nDx}/m_{w}}=\frac{\frac{\left(m_{nDx,b}\;+\;m_{nDx}\right)/m_{b}}{1\;+\;m_{w}/m_{b}}}{m_{nDx}/m_{w}}<\frac{\frac{m_{nDx,b}\;+\;m_{nDx}}{m_{b}}}{m_{nDx}/m_{w}}$$

In summary, a systematic analysis through a synthetic biofilm model adds to the toolkit of biointerface studies. Size exclusion has been replicated in alginate [41] and now in nanocellulose. While attachment and volume-exclusion were not replicated, future work using similar matrices should also consider concentration of microspheres within the nanocellulose matrix. For example, there may be a critical concentration of microspheres or bacterial cells necessary for some of the absorption effects. While these can be studied with non-toxic nanomaterials, these affects are often very difficult to uncouple in living systems [67].

## 5. Conclusion

This study aimed to emulate key physicochemical barriers to diffusion found in natural biofilms using tunable synthetic microcap biofilm matrix system. Through the controlled exposure of nanodextrans with varying size and surface charge, we evaluated the system’s ability to emulate three core physicochemical features often implicated in biofilm-associated transport resistance: size exclusion, charge interactions, and volume exclusion. The results demonstrated a statistically significant size-exclusion effect, confirming the ability of the nanocellulose-based microcaps to mimic the selective permeability of hydrated biofilm matrices. However, the designed system did not display statistically significant volume-exclusion or attachment effects, suggesting that the current microsphere concentration and charge configurations were insufficient to replicate these additional features.

These findings reflect patterns observed in natural biofilms studies, where size-based diffusion hinderance is commonly reported, but charge-based interaction and volume exclusion are more context-dependent. The absence of strong attachment or volume exclusion effects may be due to insufficient microsphere loading, incomplete charge immobilization, or the dynamic behavior of the nanodextrans in the hydrated mesh.

Future studies should explore increased microsphere loading, use of covalently bound attachment sites or incorporation of more biologically relevant surface chemistries to better recapitulate these additional transport-limiting features. Ultimately, refining this synthetic biofilm platform will enhance its utility in advancing the ecotoxicology of engineered nanomaterials.

## Supplementary Material

Supplement 1

## Figures and Tables

**Figure 3:**
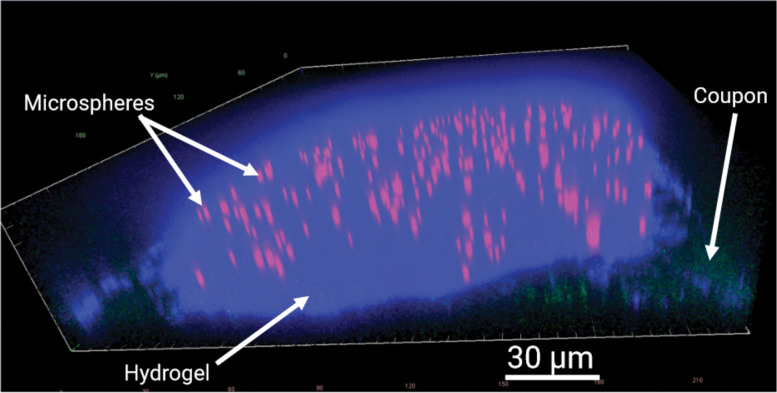
Labelled Microcap from Characterization Image. Red channel: fluorescent polystyrene microspheres (carboxyl-modified). Blue channel: CW-stained nanocellulose hydrogel. Green-channel: polycarbonate coupon-microcap interface.

**Figure 4:**
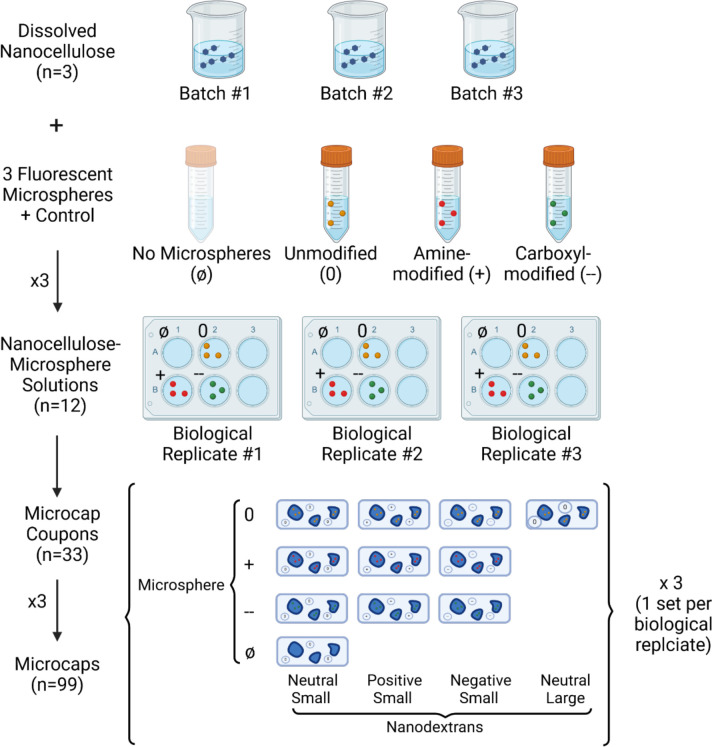
Design of Experiments and Replicates

**Table 1:** Nanodextran Characterization.

Nanodextran	Nanodextran Molecular Weight [kDa]	Size via NTA [nm]	Size via DLS [nm]	Charge via ELS [mV]
FITC-Dx-150	150	226 ± 14	115 ± 24	−1.2 ± 0.4
FITC-DEAE-150	150	178 ± 5	107 ± 16	−0.4 ± 0.4
FITC-CM-150	150	173*	771 ± 1000	−5.7 ± 3.2
FITC-Dx-2000	2000	297 ± 18 306 ± 24	81 ± 0.1	−2.4 ± 0.7

All nanodextrans dispersed in Dulbecco’s Phosphate Buffer Solution (PBS). NTA error reported as standard error from measured particle size distribution (n=5).

* -Standard error not reported on instrument. FITC-Dx-2000 size measured twice on NTA, both sizes reported. DLS/ELS error reported as standard deviation of three triplicate measurements.

**Table 2:** Summary of literature on bioaccumulation and bioconcentration kinetic parameters of ENMs in periphytic biofilms.

Type	Biofilm	Size (nm)	Hydropathy	Charge	t_C_	pBCF	Study
Latex carboxylate	Wetland	100	Hydrophilic	(−)	< 24 h	~2	[60]
Gold	Estuary	65	Hydrophobic	(+)	< 12 d	2.18	[61]
Gold polystyrene sulfonate	Estuary	50	Hydrophobic*	−53 mV	~12 h	3.46	[22]
Citrate capped silver	Estuary	30, 115	Hydrophobic	(−)	~12 h	1.4 – 1.5	[62]
Citrate capped silver	Marine	100 – 700	Hydrophobic	−18 mV	< 24 h	2.05	[47]
PVP capped silver	Benthic	117	Hydrophilic	−17 mV	>10 min, < 4 d	4.6	[63]
Titanium dioxide	Algal	150	n.d.*	−17 mV	< 28 d	4 – 5.6	[64]
Copper oxide	Periphyton	100 – 800	Hydrophilic*	−35 – +15 mV	~50 min	3.6 – 4.25	[65]
Citrate capped silver	*Aquabacterium citratiphilum*	30, 70	Hydrophobic	−60 mV	< 20 h	0.8	[66]
FITC-Dx-150	Hydrogel	92 – 240	Hydrophilic	−1.2 ± 0.4 mV	24 min	0.153	
FTIC-Dx-2000	Hydrogel + neutral spheres	81 – 330	Hydrophilic	−2.4 ± 0.7 mV	24 min	0.319	
FITC-Dx-150	Hydrogel + neutral spheres	92 – 240	Hydrophilic	−1.2 ± 0.4 mV	24 min	0.164	
FITC-DEAE-150	Hydrogel + neutral spheres	91 – 183	Hydrophilic	−0.4 ± 0.4 mV	24 min	0.132	
FITC-CM-150	Hydrogel + neutral spheres	173 – 1000	Hydrophilic	−5.7 ± 3.2 mV	24 min	0.160	
FITC-Dx-150	Hydrogel + carboxyl (−) spheres	92 – 240	Hydrophilic	−1.2 ± 0.4 mV	24 min	0.145	
FITC-DEAE-150	Hydrogel + carboxyl (−) spheres	91 – 183	Hydrophilic	−0.4 ± 0.4 mV	24 min	0.179	
FITC-CM-150	Hydrogel + carboxyl (−) spheres	173 – 1000	Hydrophilic	−5.7 ± 3.2 mV	24 min	0.135	
FITC-Dx-150	Hydrogel + amino (+) spheres	92 – 240	Hydrophilic	−1.2 ± 0.4 mV	24 min	0.125	
FITC-DEAE-150	Hydrogel + amino (+) spheres	91 – 183	Hydrophilic	−0.4 ± 0.4 mV	24 min	0.158	
FITC-CM-150	Hydrogel + amino (+) spheres	173 – 1000	Hydrophilic	−5.7 ± 3.2 mV	24 min	0.068	

n.d. no data reported.

* Some hydropathies are inferred. Titanium dioxide hydropathy depends on crystal phase which also modifies its toxicity [59].

## References

[1] Vattulainen I. and Mouritsen O.G., Diffusion in Membranes, in Diffusion in Condensed Matter, Heitjans P. and Kärger J., Editors. 2005, Springer: Berlin.

[2] Dingari N.N. and Buie C.R., Theoretical investigation of bacteria polarizability under direct current electric fields. Langmuir, 2014. 30(15): p. 4375–84. 10.1021/la500274h 24665921

[3] Van Wey A.S., , Anisotropic nutrient transport in three-dimensional single species bacterial biofilms. Biotechnol Bioeng, 2012. 109(5): p. 1280–92. 22124974 10.1002/bit.24390

[4] Prince J. and Jones A.D. 3rd, Heterogenous biofilm mass-transport model replicates periphery sequestration of antibiotics in Pseudomonas aeruginosa PAO1 microcolonies. Proc Natl Acad Sci U S A, 2023. 120(47): p. e2312995120. 37956290 10.1073/pnas.2312995120 PMC10666002

[5] Flemming H.C. and Wingender J., The biofilm matrix. Nat Rev Microbiol, 2010. 8(9): p. 623–33. 20676145 10.1038/nrmicro2415

[6] Miller M.B. and Bassler B.L., Quorum sensing in bacteria. Annu Rev Microbiol, 2001. 55: p. 165–99. 11544353 10.1146/annurev.micro.55.1.165

[7] Xu K.D., , Spatial physiological heterogeneity in Pseudomonas aeruginosa biofilm is determined by oxygen availability. Appl Environ Microbiol, 1998. 64(10): p. 4035–9. 9758837 10.1128/aem.64.10.4035-4039.1998 PMC106596

[8] Martin M., , Laboratory Evolution of Microbial Interactions in Bacterial Biofilms. J Bacteriol, 2016. 198(19): p. 2564–71. 27044625 10.1128/JB.01018-15 PMC5019067

[9] Tremblay P.L., , A genetic system for Geobacter metallireducens: role of the flagellin and pilin in the reduction of Fe(III) oxide. Environ Microbiol Rep, 2012. 4(1): p. 82–8. 23757233 10.1111/j.1758-2229.2011.00305.x

[10] Hochvaldova L., , E. coli and S. aureus resist silver nanoparticles via an identical mechanism, but through different pathways. Commun Biol, 2024. 7(1): p. 1552. 39572718 10.1038/s42003-024-07266-3 PMC11582817

[11] Secor P.R., , Staphylococcus aureus Biofilm and Planktonic cultures differentially impact gene expression, mapk phosphorylation, and cytokine production in human keratinocytes. BMC Microbiol, 2011. 11: p. 143. 21693040 10.1186/1471-2180-11-143 PMC3146417

[12] Ciofu O., , Tolerance and resistance of microbial biofilms. Nat Rev Microbiol, 2022. 20(10): p. 621–635. 35115704 10.1038/s41579-022-00682-4

[13] Campos J.V., , Advancing Nanotechnology: Targeting Biofilm-Forming Bacteria with Antimicrobial Peptides. BME Front, 2025. 6: p. 0104. 40041091 10.34133/bmef.0104 PMC11876546

[14] Mohanta Y.K., , Nanotechnology in combating biofilm: A smart and promising therapeutic strategy. Front Microbiol, 2022. 13: p. 1028086. 36938129 10.3389/fmicb.2022.1028086 PMC10020670

[15] Choi V., , Drug delivery strategies for antibiofilm therapy. Nat Rev Microbiol, 2023. 21(9): p. 555–572. 37258686 10.1038/s41579-023-00905-2

[16] Klaper R.D., The Known and Unknown about the Environmental Safety of Nanomaterials in Commerce. Small, 2020. 16(36). 10.1002/smll.202000690 32407002

[17] Suhendra E., , A Review on the Environmental Fate Models for Predicting the Distribution of Engineered Nanomaterials in Surface Waters. International Journal of Molecular Sciences, 2020. 21(12). 10.3390/ijms21124554 PMC7349326 32604975

[18] Patil S.S. and Lekhak U.M., Toxic effects of engineered carbon nanoparticles on environment. Carbon Nanomaterials for Agri-Food and Environmental Applications, 2020: p. 237–260.

[19] Nguyen M.K., Moon J.Y., and Lee Y.C., Microalgal ecotoxicity of nanoparticles: An updated review. Ecotoxicology and Environmental Safety, 2020. 201. 10.1016/j.ecoenv.2020.110781 32497816

[20] Lekamge S., , The Toxicity of Nanoparticles to Organisms in Freshwater. Reviews of Environmental Contamination and Toxicology, Vol 248, 2020. 248: p. 1–80. 30413977 10.1007/398_2018_18

[21] Salieri B., , Fate modelling of nanoparticle releases in LCA: An integrative approach towards “USEtox4Nano”. Journal of Cleaner Production, 2019. 206: p. 701–712.

[22] Burns J.M., , Surface charge controls the fate of Au nanorods in saline estuaries. Environmental Science & Technology, 2013. 47(22): p. 12844–12851. 24144224 10.1021/es402880u

[23] Peulen T.O. and Wilkinson K.J., Diffusion of Nanoparticles in a Biofilm. Environmental Science & Technology, 2011. 45(8): p. 3367–3373. 21434601 10.1021/es103450g

[24] Morrow J.B., Arango C., and Holbrook R.D., Association of Quantum Dot Nanoparticles with Biofilm. Journal of Environmental Quality, 2010. 39(6): p. 1934–1941. 21284290 10.2134/jeq2009.0455

[25] Guiot E., , Heterogeneity of diffusion inside microbial biofilms determined by fluorescence correlation spectroscopy under two-photon excitation. Photochemistry and Photobiology, 2002. 75(6): p. 570–578. 12081317 10.1562/0031-8655(2002)075<0570:hodimb>2.0.co;2

[26] Tripathi S., Champagne D., and Tufenkji N., Transport Behavior of Selected Nanoparticles with different Surface Coatings in Granular Porous Media coated with Biofilm. Environmental Science & Technology, 2012. 46(13): p. 6942–6949. 22148225 10.1021/es202833k

[27] Desmau M., , Dynamics of silver nanoparticles at the solution/biofilm/mineral interface. Environmental Science-Nano, 2018. 5(10): p. 2394–2405.

[28] Sahle-Demessie E. and Tadesse H., Kinetics and equilibrium adsorption of nano-TiO particles on synthetic biofilm. Surface Science, 2011. 605(13–14): p. 1177–1184.

[29] Thuptimdang P., Limpiyakorn T., and Khan E., Dependence of toxicity of silver nanoparticles on biofilm structure. Chemosphere, 2017. 188: p. 199–207. 28886554 10.1016/j.chemosphere.2017.08.147

[30] Li X.N., , Control of nanoparticle penetration into biofilms through surface design. Chemical Communications, 2015. 51(2): p. 282–285. 25407407 10.1039/c4cc07737g PMC4258172

[31] Ikuma K., , Deposition of nanoparticles onto polysaccharide-coated surfaces: implications for nanoparticle-biofilm interactions. Environmental Science-Nano, 2014. 1(2): p. 117–122.

[32] Liang Y., , Interaction forces between colloidal particles in liquid: Theory and experiment. Advances in Colloid and Interface Science, 2007. 134-35: p. 151–166. 17499205 10.1016/j.cis.2007.04.003

[33] Nevius B.A., , Surface-functionalization effects on uptake of fluorescent polystyrene nanoparticles by model biofilms. Ecotoxicology, 2012. 21(8): p. 2205–2213. 22806556 10.1007/s10646-012-0975-3

[34] Habimana O., , Diffusion of Nanoparticles in Biofilms Is Altered by Bacterial Cell Wall Hydrophobicity. Applied and Environmental Microbiology, 2011. 77(1): p. 367–368. 21037304 10.1128/AEM.02163-10 PMC3019707

[35] Schambeck C.M., , Chemical and physical properties of alginate-like exopolymers of aerobic granules and flocs produced from different wastewaters. Bioresour Technol, 2020. 312: p. 123632. 32531737 10.1016/j.biortech.2020.123632

[36] Leid J.G., , The exopolysaccharide alginate protects Pseudomonas aeruginosa biofilm bacteria from IFN-gamma-mediated macrophage killing. J. Immunol., 2005. 175(11): p. 7512–7518. 16301659 10.4049/jimmunol.175.11.7512

[37] Felz S., , Impact of metal ions on structural EPS hydrogels from aerobic granular sludge. Biofilm, 2020. 2: p. 100011. 33447798 10.1016/j.bioflm.2019.100011 PMC7798472

[38] Moraes Schambeck C., Ribeiro da Costa R.H., and Derlon N., Phosphate removal from municipal wastewater by alginate-like exopolymers hydrogels recovered from aerobic granular sludge. Bioresour Technol, 2021. 333: p. 125167. 33894450 10.1016/j.biortech.2021.125167

[39] Taylor D.W. and Jones A.A.D., Synthesis of Metal-Modified Nanocellulose as a Biofilm Analogue for Biofilm Mimicry in Biomedical and Environmental Applications. Biopolymers, 2025. 116(4). 10.1002/bip.70029 PMC12415988 40446082

[40] Wang L.S., Gupta A., and Rotello V.M., Nanomaterials for the Treatment of Bacterial Biofilms. Acs Infectious Diseases, 2016. 2(1): p. 3–4. 27622944 10.1021/acsinfecdis.5b00116 PMC5848070

[41] Rodriguez-Suarez J.M., , Heterogeneous Diffusion of Polystyrene Nanoparticles through an Alginate Matrix: The Role of Cross-linking and Particle Size. Environ Sci Technol, 2020. 54(8): p. 5159–5166. 32182039 10.1021/acs.est.9b06113

[42] Jones A.A.D. and Buie C.R., Continuous shear stress alters metabolism, mass-transport, and growth in electroactive biofilms independent of surface substrate transport. Scientific Reports, 2019. 9(1): p. 2602. 30796283 10.1038/s41598-019-39267-2 PMC6385357

[43] Herbert-Guillou D., Tribollet B., and Festy D., Influence of the hydrodynamics on the biofilm formation by mass transport analysis. Bioelectrochemistry, 2001. 53(1): p. 119–25. 11206919 10.1016/s0302-4598(00)00121-5

[44] Sankaran J., , Single microcolony diffusion analysis in Pseudomonas aeruginosa biofilms. NPJ Biofilms Microbiomes, 2019. 5(1): p. 35. 31728202 10.1038/s41522-019-0107-4 PMC6841743

[45] Zhang Z., Nadezhina E., and Wilkinson K.J., Quantifying diffusion in a biofilm of Streptococcus mutans. Antimicrob Agents Chemother, 2011. 55(3): p. 1075–81. 21189346 10.1128/AAC.01329-10 PMC3067071

[46] Stewart P.S., Diffusion in biofilms. J Bacteriol, 2003. 185(5): p. 1485–91. 12591863 10.1128/JB.185.5.1485-1491.2003 PMC148055

[47] Fabrega J., , Impact of silver nanoparticles on natural marine biofilm bacteria. Chemosphere, 2011. 85(6): p. 961–966. 21782209 10.1016/j.chemosphere.2011.06.066

[48] Gil-Allué C., , Long-term exposure to silver nanoparticles affects periphyton community structure and function. Environmental Science-Nano, 2018. 5(6): p. 1397–1407.

[49] MacCuspie R.I., , Challenges for physical characterization of silver nanoparticles under pristine and environmentally relevant conditions. J Environ Monit, 2011. 13(5): p. 1212–26. 21416095 10.1039/c1em10024f

[50] Carmonaribeiro A.M. and Midmore B.R., Synthetic Bilayer Adsorption onto Polystyrene Microspheres. Langmuir, 1992. 8(3): p. 801–806.

[51] Singh R., , Penetration barrier contributes to bacterial biofilm-associated resistance against only select antibiotics, and exhibits genus-, strain- and antibiotic-specific differences. Pathog Dis, 2016. 74(6). 10.1093/femspd/ftw056 27402781

[52] Prince J., Theoretical, Simulation, and Experimental Approaches to Understanding Periphery Sequestration of Diffusing Chemical Species in Bacterial Biofilms as a Mechanism for Antibiotic Recalcitrance. 2024, Duke University: Durham, NC.

[53] Kurniawan A., , Characterization of the internal ion environment of biofilms based on charge density and shape of ion. Colloids Surf B Biointerfaces, 2015. 136: p. 22–6. 26350802 10.1016/j.colsurfb.2015.08.047

[54] Amsden B., Solute Diffusion within Hydrogels. Mechanisms and Models. Macromolecules, 1998. 31(23): p. 8382–8395.

[55] Bryers J.D. and Drummond F., Local Macromolecule Diffusion Coefficients in Structurally Non-Uniform Bacterial Biofilms Using Fluorescence Recorvery After Photobleaching, in Biotechnol Bioeng. 1998. p. 462–473. 10099452

[56] Park R.A., Clough J.S., and Wellman M.C., AQUATOX: Modeling environmental fate and ecological effects in aquatic ecosystems. Ecological Modelling, 2008. 213(1): p. 1–15.

[57] Hobbs W.O., , Toxic Burdens of Freshwater Biofilms and Use as a Source Tracking Tool in Rivers and Streams. Environmental Science & Technology, 2019. 53(19): p. 11102–11111. 31460753 10.1021/acs.est.9b02865

[58] Luoma S.N. and Rainbow P.S., Why is metal bioaccumulation so variable? Biodynamics as a unifying concept. Environmental Science & Technology, 2005. 39(7): p. 1921–1931. 15871220 10.1021/es048947e

[59] Bolis V., , Hydrophilic/hydrophobic features of TiO2 nanoparticles as a function of crystal phase, surface area and coating, in relation to their potential toxicity in peripheral nervous system. J Colloid Interface Sci, 2012. 369(1): p. 28–39. 22209580 10.1016/j.jcis.2011.11.058

[60] Flood J.A. and Ashbolt N.J., Virus-sized particles can be entrapped and concentrated one hundred fold within wetland biofilms (Reprinted from Advances in Environmental Research, vol 3, pg 403–411, 2000). Advances in Environmental Research, 1999. 3(4).

[61] Ferry J.L., , Transfer of gold nanoparticles from the water column to the estuarine food web. Nature Nanotechnology, 2009. 4(7): p. 441–444. 10.1038/nnano.2009.157 19581897

[62] Cleveland D., , Pilot estuarine mesocosm study on the environmental fate of Silver nanomaterials leached from consumer products. Science of the Total Environment, 2012. 421: p. 267–272. 22369864 10.1016/j.scitotenv.2012.01.025

[63] Kroll A., , Mixed messages from benthic microbial communities exposed to nanoparticulate and ionic silver: 3D structure picks up nano-specific effects, while EPS and traditional endpoints indicate a concentration-dependent impact of silver ions. Environmental Science and Pollution Research, 2016. 23(5): p. 4218–4234. 26122573 10.1007/s11356-015-4887-7 PMC4766215

[64] Wright M.V., , Titanium dioxide nanoparticle exposure reduces algal biomass and alters algal assemblage composition in wastewater effluent-dominated stream mesocosms. Science of the Total Environment, 2018. 626: p. 357–365. 29351883 10.1016/j.scitotenv.2018.01.050

[65] Miao L.Z., , Effects of pH and natural organic matter (NOM) on the adsorptive removal of CuO nanoparticles by periphyton. Environmental Science and Pollution Research, 2015. 22(10): p. 7696–7704. 25510615 10.1007/s11356-014-3952-y

[66] Grün A.Y., , Sublethal concentrations of silver nanoparticles affect the mechanical stability of biofilms. Environmental Science and Pollution Research, 2016. 23(23): p. 24277–24288. 27650851 10.1007/s11356-016-7691-0

[67] Fabrega J., Renshaw J.C., and Lead J.R., Interactions of silver nanoparticles with Pseudomonas putida biofilms. Environ Sci Technol, 2009. 43(23): p. 9004–9. 19943680 10.1021/es901706j

