# Supplementary material for: Nanocellulose hydrogels as bio-interface analogs for studying nanomaterial transport and accumulation
Source: bioRxiv. 2026 Feb 4:2026.02.02.703274. Preprint. [Version 1] doi: 10.64898/2026.02.02.703274 (PMC12889593; doi:10.64898/2026.02.02.703274)
Supplement: Supplement 1 [file media-1.pdf]

Supplementary Information Nanocellulose hydrogels as bio-interface analogs for studying nanomaterial transport and accumulation

Joshua Prince[1,a], Darryl Taylor[2,b] and A-Andrew D. Jones, III[1,2,3,c,*]

Affiliations:

1. Department of Civil & Environmental Engineering, Pratt School of Engineering, Duke University, Durham, NC 27708
2. University Program in Materials Science & Engineering, Duke University, Durham, NC 27708,
3. Thomas Lord Department of Mechanical Engineering & Materials Science, Pratt School of Engineering, Duke University, Durham, NC 27708
    a. Present address: joshuaprince58@gmail.com, ICF ORCID: 0000-0001-6050-9544
    b. darryl.taylor@duke.edu, ORCID: 0000-0001-6525-857X
    c. Corresponding author: andrew.jones3@duke.edu, ORCID: 0000-0003-3840-8039

Nanoparticle Characterization

Three methods were used to analyze the nanodextrans in the study. The first was Nanoparticle Tracking Analysis (NTA), performed using a Malvern NanoSight Pro with a 532 green laser, which detects particle size using light microscopy to track individual particle movements. The second was Dynamic Light Scattering (DLS), performed using a Anton-Paar Litsizer 500 Particle Analyzer, which detects particle size using fluctuations in light signal through a sample due to Brownian motion. The final was Electrophoretic Light Scattering (ELS), which detects particle surface charge using fluctuations in light signal through a sample due to electrophoresis.

All samples were prepared by taking dry powder formulations of the nanodextrans and dispersing them in Dulbecco's Phosphate Buffer Solution (0.137 M sodium chloride (CAS# 7647145, ACS reagent grade ≥99.0% Sigma-Aldrich), 0.0027 M potassium chloride (CAS# 7447407, ACS reagent grade 99.0 – 100.5%, Sigma-Aldrich), 0.01 M sodium phosphate dibasic (CAS# 7558794, bioreagent grade ≥99.0% Sigma-Aldrich), 0.0018 M potassium phosphate monobasic (CAS# 7778770, ≥99.0%, Sigma-Aldrich).

For DLS, 1 mL of sample was added to a plastic cuvette and analyzed. Instrument settings were a refractive index of 1.45 for the nanodextran, a refractive index of 1.33 for the PBS solution, 1 minute of equilibration time, a temperature of 25°C, back-scattering, automatic filters, automatic focus, automatic number and length of runs, and automatic analysis modules to infer diffusivity and hydrodynamic diameter distribution from generated autocorrelation functions. All samples were ran in triplicate.

For ELS, 0.600 mL of sample was added to a zeta-potential Omega cuvette (#225288) and analyzed. Instrument settings were a PBS solvent, a temperature of 25°C, 1 minute of equilibration time, automatic power adjustments, automatic number of runs, and zeta-potential inferred using the Smoluchowski relation with a Henry Factor of 1.5. All samples were run in triplicate.

For NTA, samples were analyzed using automatic equipment settings. 1 mL of sample was analyzed using a constant flowrate of 3 µL/min, with 5 replicates measurements per sample.

Materials and Equipment

The following chemicals were used in this experimental process:

Cellulose powder (CAS#9004-34-6 | Sigma-Aldrich | Burlington, MA);

Sodium hydroxide (CAS#1310-73-2 | Sigma-Aldrich | Burlington, MA);
Urea (CAS#57-13-6 | Sigma-Aldrich | Burlington, MA)
Sodium chloride (CAS# 7647145, ACS reagent grade ≥99.0% Sigma-Aldrich | Burlington, MA)
Potassium chloride (CAS# 7447407, ACS reagent grade 99.0 – 100.5%, Sigma-Aldrich | Burlington, MA)
Sodium phosphate dibasic (CAS# 7558794, bioreagent grade ≥99.0% Sigma-Aldrich | Burlington, MA)
Potassium phosphate monobasic (CAS# 7778770, ≥99.0%, Sigma-Aldrich)

Polystyrene microspheres (Spherotech | Lake Forest, IL) with no surface modifications (Prod# FP-0862-2),
Polystyrene microspheres carboxylate-modified (Prod# FP-0862-2)
Polystyrene microspheres amine-modified (Prod# FP-0862-2)
Fluorescent nanodextrans (TdB Labs, Uppsala, Sweden) with no chemical modification
Fluorescent nanodextrans lower molecular weight (Prod# FITC-Dx-150)
Fluorescent nanodextrans carboxymethyl groups (Prod# FITC-CM-dextran-150)
Fluorescent nanodextrans diethylaminoethyl groups (Product # FITC-DEAE-dextran-150)
Fluorescent nanodextrans with no chemical modification and high molecular weight (Prod# FITC-Dx-2000)
Calcofluor White (CW) stain (Cat# 29067 | Biotium | Fremont, CA, USA).

The following materials were used in this experimental process:
6-well culture plate (Pro# 10062-892 | VWR | Radner, PA, USA)
5 mL syringe with Leur-lock tip (SKU# 309646)
27G nozzle (SKU# NZ3270005001 | CELLLINK | Gothenburg, Sweden)
two-channel microscopy flow cell (Prod# FC 275-AL | BioSurface Technologies | Bozeman, MT, USA) with 6x25x2 mm polycarbonate coupons
1/8" ID PVC tubing (Prod# 13-712-77 | Fisher | Pittsburgh, PA).

The following equipment was used in this process:
Refrigerated centrifuge with fixed angle rotor (Prod# 5910 Ri | Eppendorf | Hamburg, Germany);
Pressure-driven microfluidic system (Fluigent | Lowell, MA) with a primary pressure controller (Prod# LU-FEZ-0345), valve controller (Prod# ELU-SEZ), computer link (Prod# LU-LNK-0002), flowrate sensor/controller (Prod# FLU-L-D), multi-switch valve (Prod# ESSMSW003) connected via 0.020" ID 1/16" OD tubing (Prod# CTQ-KIT-HQ)
Zeiss LSM 900 confocal microscope (Zeiss | Oberkochen, Germany)
NanoSight Pro with NTA Technology (Model # HBG5000 | Malvern Panalytical | Malvern, United Kingdom)
Anton-Paar Particle Analyzer Litesizer 500 (Anton-Paar | Graz, Austria)

Image Analysis MATLAB Code
Also available on Github https://github.com/Dr-Jones-SEEL-Team/Bioaccumulation_NP

Below is an example of the code used to process the z-stack imaging data in MATLAB. Separate code as outlined in the Main Body was used for ImageJ processing.

```
Nanodextran Accumulation in Nanocellulose Time-Lapse Imaging Data Analyzer-

Refresh MATLAB Environment
clc
close all
clear all
Launch Parellel Computing Pool
% parpool('local',10); %Enable parellel pool
Change MATLAB path and Add bfmatlab directory to path
% % Josh's Mac homepath
% homepath='/Users/joshuaprince/Library/CloudStorage/Box-Box/Quantum
Biofilms/Processed Data/Synthetic-Quantum Biofilms/JP_190/JP_177_Chan1_Microcap1';
%Define home directory for code
% cd(homepath) %Change directory to this home path

% Josh's PC homepath
homepath='C:\Users\joshu\Box\Quantum Biofilms\Processed Data\Synthetic-Quantum
Biofilms\JP_218\JP_218_Microcap1'; %Define home directory for code
cd(homepath) %Change directory to this home path

bfmatlabdir='bfmatlab'; %Specify directory to add
addpath(genpath(bfmatlabdir)); %Add the directory

functionscriptdir='subfunctions_v3'
addpath(genpath(functionscriptdir)); %Add directory
Analysis of Characterization Image
Read in the Characterization/Accumulation/Desorption Image
% file_path1='JP_190_Nanocellulose_Plain-PS_FITC-DEAE-150_20240707_Confocal_1.czi'
%Load in Characterization Image
file_path2='JP_218_Nanocellulose_Plain-PS_FITC-DEAE-150_20240719_Confocal_5.czi'
%Load in Accumulation Image
% file_path3='JP_177_Nanocellulose_Amino-PS_FITC-DEAE-
150_20240413_Confocal_9.czi' %Load in Desorption Image
% file_paths={file_path1;file_path2;file_path3 ;
Retrieve relevant metadata
reader = bfGetReader(file_path2);
metadata = retrieve_metadata(file_path2);
numX=metadata(1); % image width, pixels
numY=metadata(2); % image width, pixels
numZ=metadata(3); % number of Z slices
numC=metadata(4); % number of channels
numP=metadata(5); % number of total planes
```

```
numI=metadata(6); % number of total images
locZ1=metadata(7); %Get top z-position (yes, everything is inverted in Z, might fix in post, might not, whatever)
locZ2=metadata(8); %Get bottom z-position (yes, everything is inverted in Z, might fix in post, might not, whatever)
locXc=metadata(9); %Get z-stack x-position (Center)
locYc=metadata(10); %Get z-stack y-position (Center)
sizeX=metadata(11); %Get the area covered by X-pixel
sizeY=metadata(12); %Get the area covered by X-pixel
sizeZ=metadata(13); %Get the area covered by X-pixel
wavelengthC1=metadata(14); %Get the emission wavelength for Channel 1
wavelengthC2=metadata(15); %Get the emission wavelength for Channel 2
wavelengthC3=metadata(16); %Get the emission wavelength for Channel 3

scaling= [sizeX,sizeY,sizeZ]; %Define the size of pixels in each spatial dimension
scaling=double(scaling); %Convert between MATLAB data formats
scaling_base=min(scaling);  %Define the base of the new scaling vector
for i=1:length(scaling)
   scaling(i)=round(scaling(i)/scaling_base,0);
end
zfactor=scaling(3); %Define factor needed to rescale the z-direction
%Leave room for other metadata I need to retrieve
```

Create Master Pixel-Value Data Matrices

```
for j=1:numC %Loops through number of channels on the file
   master_pixel_generator(file_path2,numX,numY,numZ,j); %Generate master pixel value matrix for Channel 1
end
```

So, after trying to just load in the data as is into a giant array, it is pretty clear that this will be too much of a drain on memory. Which makes sense. These are large arrays of data. So, I'll have to be more clever about only ever calling subsets of data when using it. Should be possible.

After moving over to running this script on a DCC virtual machine with boatloads of memory (but probably not enough CPUs, only 4), I was able to create these large data matrices.

Segmenting Microspheres Channel

Goal is to generate a binary file partitioning pixel data matrixinto "in-microsphere" volume (binary value 1) and "non in-microspeheres volume" (binary value 0).

```
datafilename=sprintf('datac3.mat');
microsphere_segmentation(datafilename)
```

Segmentation of Channel 2: Hydrogel

```
% Convert ImageJ  segementations into MATLAB .mat datafiles
coupon_zposition=ImageJ_convert(file_path2) %Save the z-plane with the largest amount of segmented hydrogel, use as coupon interface
```

Segmentation of Channel 1: Coupon

```
% Segment Coupon
```

```
datafilename=sprintf('datac1.mat');
coupon_segmenter(datafilename,coupon_zposition); %Create coupon volume below coupon
position

% Remove microsphere volume from hydrogel segmentation
datafilename_hydrogel=sprintf('datac2_ImageJsegmented.mat');

%Remove coupon volume from hydrogel segementation
datafilename_coupon=sprintf('datac1_couponsegmented.mat');
mask_remover_binary(datafilename_hydrogel,datafilename_coupon)
```

Define water volume

```
datafilename_coupon=sprintf('datac1_couponsegmented.mat');
datafilename_microspheres=sprintf('datac3_OTSUed.mat');
datafilename_hydrogel=sprintf('datac2_ImageJsegmented_removebinarymask.mat');
water_segmenter(datafilename_hydrogel,datafilename_microspheres,datafilename_coupon)
%Define water volume

% 3-D viewer of water segmentation

datafilename=sprintf('water.mat')
downF=2;
new_numX=round(numX/downF,0);
new_numY=round(numY/downF,0);
new_numZ=round(numZ/downF,0);
zstack_resizer(datafilename,new_numX,new_numY,new_numZ)
datafilename_resized=sprintf('water_resized.mat');
threeD_rendering(datafilename,scaling) %3-D Render the segemented volume in
volumeViewer
```

Initialize Output Vector

```
%Define the three entries in the hdyrogel vector
stages={'Accumulation' ;

%Define Image Phases of Interest
phases={'Microcap';'Hydrogel';'Microspheres';'Water';'Coupon' ;

%Define variable types for table
varTypes={'double','double','double','double','double' ;

%Initiaite Output Table
```

```
export_table=table('Size',[1,5],'VariableTypes',varTypes,'VariableNames',phases,'RowNames',
stages)
```

Quantifying Average Channel 1 values in Hydrogel (with Microspheres)

```
datafilename_pixeldata=sprintf('datac1.mat') ;
datafilename_binarymask=sprintf('datac2_ImageJsegmented_removebinarymask.mat');
%Grab hydrogel binary mask
export_table{1,'Hydrogel' =average_value(datafilename_pixeldata,datafilename_binarymask);
```

Quantifying Average Channel 1 values in Microsphere Volume

```
datafilename_pixeldata=sprintf('datac1.mat') ;
datafilename_binarymask=sprintf('datac3_OTSUed.mat') ;
export_table{1,'Microspheres'
=average_value(datafilename_pixeldata,datafilename_binarymask);
```

Quantifying Average Channel 1 values in Water Volume

```
datafilename_pixeldata=sprintf('datac1.mat') ;
datafilename_binarymask=sprintf('water.mat') ;
export_table{1,'Water' =average_value(datafilename_pixeldata,datafilename_binarymask);
```

Quantifying Average Channel 1 values in Coupon Volume

```
datafilename_pixeldata=sprintf('datac1.mat') ;
datafilename_binarymask=sprintf('datac1_couponsegmented.mat') ;
export_table{1,'Coupon' =average_value(datafilename_pixeldata,datafilename_binarymask);
```

Export Nanodextran-concentration in phases matrix

```
writetable(export_table,'JP_218_Microcap2_v3', 'WriteRowNames',true)
```
